# Farmers, development agents, and cooperative workers' perspectives on the status, impacts, and solutions of agrochemical adulteration

**DOI:** 10.1371/journal.pone.0357311

**Published:** 2026-09-11

**Authors:** Negawo Lelissa, Tewodros Mulugeta

**Affiliations:** Department of Biology, College of Science and Mathematics Education, Kotebe University of Education, Addis Ababa, Ethiopia; University of Georgia, UNITED STATES OF AMERICA

## Abstract

The use of agricultural inputs, such as pesticides and fertilizers, has increased due to agricultural expansion. In developing countries, such as Ethiopia, the agricultural sector is experiencing an expanding demand and consumption of agrochemicals to support the ever-increasing population. The increase in land dedicated to agriculture is accompanied by a rise in agrochemical demand and consumption. The increased demand for agrochemicals may lead to the distribution of low-quality, adulterated, or counterfeit products. To prevent crop loss and minimize the environmental and economic impacts of adulterated products on farmers, it is crucial to investigate their understanding of agrochemical adulteration, its prevalence, and its status. Therefore, this study aimed to assess farmers and agricultural workers, such as field agents and cooperative workers, perspectives on the incidence and impact of agrochemical adulteration in the Wolmera district, located 29 km west of Addis Ababa. Using a cross-sectional design, data were collected from 384 participants through structured interviews. According to the findings, respondents confirmed the existence of adulterated agrochemicals in their local markets, with herbicides, fungicides, and insecticides identified as the most commonly adulterated products. The types of adulteration reported included product dilution and reduced weight. Respondents indicated significant negative impacts, including crop damage, yield reduction, and financial loss, which ultimately eroded farmers’ trust in agrochemical usage. To address these challenges, an adulteration regulation mechanism should be in place, the use of technology to identify adulterated or counterfeit products should be encouraged, and accountability for fraud in the value chain should be ensured.

## Introduction

Meeting the food demand of the ever-growing world population requires an effective and efficient agricultural system that can enhance productivity and produce sufficient food [1]. To boost productivity, various agricultural inputs are utilized, including improved seeds and agrochemicals, such as pesticides and fertilizers [2]. Over the past five decades, the global annual fertilizer consumption has surged to 206 million metric tons in 2024 [3]. The use of pesticides has increased productivity by one-third. Without pesticides, the production of fruits, vegetables, and cereals would fall by 78%, 54%, and 32%, respectively. In 2021 alone, the world used 3.54 million tons of pesticide active ingredients [4]. Since 2003, there has been a notable increase in the use of agrochemicals in Ethiopia [5]. The Ethiopian government imported 1.7 million tons of fertilizer in 2023, costing over $ 1 billion [6]. The percentage of crop area fertilized rose from 25% in 2003 to 60% in 2019. Similarly, the amount of fertilizer used per hectare increased from 22 kg in 2003–110 kg/hectare in 2019 [7]. Likewise, there has been a significant rise in pesticide use in Ethiopia [8]. The annual amount of pesticides imported to Ethiopia between 2016 and 2020 was close to 18,000 MT [9]. Ethiopia relies on importing agrochemicals, which places a heavy burden on end users due to price hikes and the national foreign currency reserve [10].

Agricultural inputs should contain adequate ingredients to achieve the desired outcomes. Agro-chemicals with fewer active ingredients cannot effectively reduce pests or weeds [11,12]. One reason for the deficiency of active ingredients in agricultural inputs is adulteration. Fertilizer adulteration may include the addition of sand, dirt, or salt to fertilizer bags. Similarly, pesticides can be adulterated by adding water [12]. Adulteration also includes the use of low-quality raw materials during the active ingredient production or formulation, or low-quality packaging materials [13]. According to [12], concrete evidence does not support the claims of adulteration of mineral fertilizer. However, adulteration is common in chemical pesticides. Counterfeiting is also a significant problem with agrochemicals. In counterfeiting, fertilizer bags without any fertilizing agents can be sold as fertilizer. In addition, only water or non-pesticide agents can be sold as a pesticide.

Crops treated with adulterated agrochemicals will have their growth adversely affected. Adulteration of both fertilizers and pesticides makes them ineffective and mainly affects economic profit in many ways. Applying a fertilizer or a pesticide with a less active ingredient requires a reapplication, which incurs additional cost. The productivity will also be compromised, resulting in the production of low yields per unit area [12]. The undesirable compounds added to the products could also threaten human health, the environment, and the economy [13,14]. The trade in counterfeit pesticides has significant negative economic impacts on governments, affecting sales, revenue, and employment. In 2020, illegal pesticides caused a 2.4% reduction in authentic pesticide sales in Europe [15]. The estimated global loss due to counterfeit products is 5.4 billion USD [13,16]. According to an earlier report [17], close to a third of pesticides sold in developing countries were below the international quality standard, attributed to the poor production and formulation, inadequate selection of chemicals, and a weak quality control system. This poses a threat to human health and the environment.

Due to the slow verification and registration process of new agricultural inputs like pesticides, the presence of older and harmful pesticides is very likely in many developing countries. Multinational companies often offer older pesticides, which have already recovered their development costs, at lower prices, making them appealing to low-income countries. Products that are banned in the EU are being exported to developing countries due to less strict regulations. Additionally, issues such as adulterated pesticide imports and the illegal blending and distribution of local pesticide variants have emerged, enabling the sale of cheaper products to farmers [16].

Agrochemical adulteration occurs at various stages of the supply chain, involving manufacturers, distributors, and retailers. Understanding the awareness of the user, the key stages where the adulteration occurs, the aspects of the adulteration, and the impacts in the sector is the first step in the prevention. Users’ understanding can play a crucial role in avoiding adulterated products, which ultimately leads to reduced acts of adulteration, along with strategies to mitigate the problem. Therefore, this study aimed to survey the perspectives of farmers and other agricultural stakeholders on the status of agrochemical adulteration and its impacts on the agriculture sector.

## Materials and methods

### Description of the study area

The study was conducted in Wolmera district, which is located 29 km west of Addis Ababa. The area's altitude ranges from 2,154–2,494 m.a.s.l., with an average annual rainfall between 1,200 and 2,250 mm. The climate of the region varies from Weina-dega to Dega. There are 18 kebeles in the woreda, each with one general agriculture farmers’ cooperative responsible for supplying agrochemicals to local farmers. The total land area of Wolmera is 66,550 hectares, with 44,200 hectares dedicated to agriculture, while the remaining land is used for purposes such as forestry, residential development, and investments. The population of Wolmera is 98,280, with 47,449 males and 50,831 females. Major crops cultivated in the woreda include wheat, barley, teff, beans, peas, maize, and sorghum. Annually, 40,212.5 quintals of fertilizers and 24,345 liters of pesticides are utilized in this region. Pesticide application covers around 29,875 hectares of cropland. Each kebele is equipped with a primary health care unit that offers health services to farmers. The primary pest affecting crops in the area is the cutworm, while rust is the most common disease encountered (Wolmera Woreda Agriculture Office, unpublished).

### Research design and data collection tools

This study was a cross-sectional study designed to gather data from farmers, development agents, and general agriculture farmers’ cooperative workers. Three separate, structured, closed-ended questionnaires were developed for each group of study participants. The questionnaire developed for farmers had five sections: demographic (12 items), knowledge about agrochemical adulteration (13 items), the status of adulteration (4 items), impact of agrochemical adulteration (5 items), and potential solutions (5 items). Two separate questionnaires with sixteen items each were also developed for Development Agents (DAs) and farmers’ cooperative workers. The questionnaires were developed based on the objectives of the study and the literature. The farmers’ questionnaire was tested on five farmers before being administered to the target respondents. Based on the response, a minor adjustment was made before usage. Besides, the inputs were used to modify the remaining questionnaires. It was also reviewed and validated by professionals. As a limitation, the reported results were solely dependent on the perception of the study participants and what they experienced as farmers in their farming practices.

### Sample size and sampling method

The sample size was determined using Cochran’s formula


$$\begin{array}{c} n_{\text{0}}=\frac{z^{\text{2}}.p.\;(\text{1}-p)}{e^{\text{2}}} \end{array}$$

Where $n_{\text{0}}$ = sample size for an infinite population

*Z* = Z-score (1.96 for 95% confidence level)

*p* = estimated proportion of population (0.5 if unknown)

*e* = margin of error (0.05)

N = population size (98,280)

A total of 384 respondents participated in the study, comprising 18 development agents working in the selected kebeles (3 per kebele), 12 workers from the general agriculture farmers’ cooperatives (2 per kebele), and 354 farmers. The respondents were above 18 years of age and farmers/DAs/workers of a farmers’ cooperative. The kebeles in the district were initially divided into two groups based on their climatic conditions, Dega and Woinadega. Three kebeles from Dega (Bekeka fi Kore Odo, Geba Robi, and Wolmera Choke) and three kebeles from Woindadega (Geresu Seda, Asgori, and Nano Suba) were randomly selected. The number of respondents/households was predetermined based on the population at each kebele. Respondents were selected systematically by walking through residential areas and selecting houses at random, every fifth house for households close to each other and every 3^rd^ house when households are scattered. In locations where households were widely scattered, farmers were interviewed as they were encountered in their homes along the direction of the data collector’s walking route. A total of 73, 59, 45, 67, 31, and 79 respondents were selected from Wolmera Choke, Geba Robi, Bekeka fi Kore Odo, Nano Suba, Asgori, and Geresu Seda, respectively. The data collection started on January 4^th,^ 2025, and was completed on March 29, 2025.

### Statistical analysis

JMP Pro 17 was used to analyze data. The demographic characteristics of the respondents were analyzed using descriptive statistics and an independent chi-square test. The independent chi-square test was used to analyze the association between the demographic characteristics, such as educational background, gender, training, and experience in agriculture, and the dependent variables, such as awareness of adulteration, the ability to identify adulterated agricultural inputs, the methods of identifying adulterated products, and the incidence of purchasing adulterated products. In addition, logistic regression was used to analyze the association between educational background, age, and experience in agriculture.

### Ethics declarations

This study was conducted in accordance with the Declaration of Helsinki. Verbal informed consent was obtained from all participants, and their privacy and confidentiality were respected throughout the data collection and analysis processes. Informants were informed that the interview was fully voluntary and they could leave the interview at any time. Their consent was witnessed by their willingness to fully participate in the interview. This study was reviewed and approved by the Scientific Ethical Review Committee (SERC) at Kotebe University of Education.

## Result

### Farmers

#### Demographic and socioeconomic characteristics.

A total of 354 farmers participated in the survey across six Kebeles: Asgori, Bekeka fi Kore Odo, Geba Robi, Geresu Seda, Nano Suba, and Wolmera Choke. The number of female participants was lower than that of male participants. Female participants accounted for only 13% (47/354), whereas male participants accounted for 87% (307/354). Respondents’ ages were classified into four groups, with the majority falling within the 40–49 age range (38%, 136/354), followed by the 30–39 age group (34%, 121/354). The youngest age group, 20–29, and the oldest group, 50–59, represented 7% (25/354) and 20% (72/354) of the respondents, respectively. The majority of the respondents (45%, 161/354) reported 11–20 years of experience. In total, 34%, 12%, and 8% of the farmers reported 21–30, 1–10, and 31–40 years of experience, respectively.

#### Farming and agro input usage practice.

Wheat was the main crop grown by the majority of respondents in the study area. This was followed by barley, teff, and sorghum. The majority of the respondents own land–1–5 hectares (91%/321/354), and the remaining 9% (33/354) owned 6–10 hectares of land. Regarding the respondents’ educational backgrounds, 36% (129/354) were illiterate, and 51% (179/354) and 13% (46/354) attended primary and secondary schools, respectively. Respondents accessed the land through ownership, rent, or both. The majority (57%, 201/354) of respondents acquired land through either rent or ownership. Slightly more than one-tenth of the respondents (14%, 50/354) rented, and 29% (103/354) owned a piece of land. Fertilizers, herbicides, fungicides, and insecticides were the agricultural inputs used by all the respondents (100%). All the respondents acknowledged that they had the opportunity to receive agrochemical training.

#### Agrochemical adulteration.

The study participants reported that they were aware of agrochemical adulteration 100% (354/354) and received information through formal channels (extension services and training) and informal networks (peer discussions and personal observations). In addition, all participants confirmed that they had received training on agrochemical adulteration and experienced chemical adulteration. They also reported being able to differentiate adulterated products from unadulterated ones. However, the majority of respondents (90%, 319/354) were not fully confident in their identification of adulterated products, and the remaining 10% were confident to some extent. According to the independent chi-square test result, all female respondents reported not being confident in their identification capacity of adulterated products; whereas, 11.4% of the male respondents reported being somewhat confident (Pearson χ² = 5.95, df = 1, *p* = 0.015). The logistic regression demonstrated that age, educational background, and experience in agriculture had no impact on respondents’ capacity to identify adulterated products (*p* > 0.05).

For a higher proportion of respondents (78%, 276/354), the effectiveness of the products was used to identify whether or not a product was adulterated. The way the products were packaged, and effectiveness were employed by 16% (58/354) of the respondents. Weight difference and effectiveness were the means used by 6% (20/354) of the respondents to identify adulterated products. Independ chi-square test indicated that the educational background significantly determined the method used to differentiate adulterated agrochemicals (Pearson χ² = 10.983, df = 4, *p* = 0.027). The majority of the illiterate farmers (86.0%) relied on product effectiveness.

**Fig 1:**
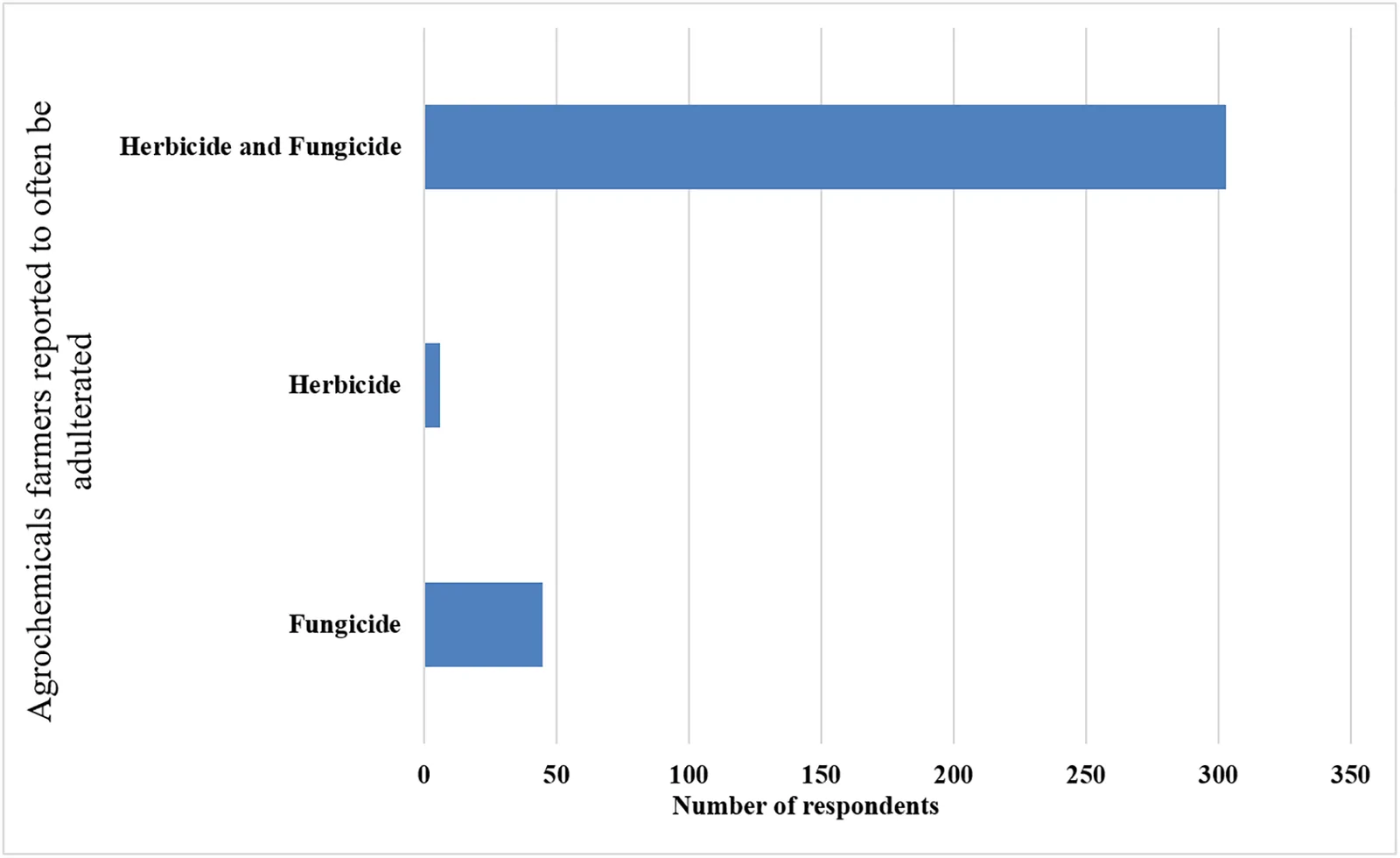
Agrochemical products that farmers have reported as being frequently adulterated or compromised in quality.

All participants also confirmed that they had used an adulterated product, mistakenly thinking that it was genuine (100%, 354/354). The dilution of products and reduced weight were the types of adulteration that respondents reported encountering. The majority of the respondents encountered both dilution and weight reduction types of adulteration. Dilution and weight reduction were reported by 14% and 6% of the respondents, respectively. All respondents reported that they sometimes bought adulterated products. Fungicides and herbicides were more susceptible to adulteration in the study area. A significant proportion of respondents reported both herbicides and fungicides as the most adulterated products (χ2 = 441, p < 0.0001) (Fig 1).

All the respondents (354/354) reported the presence of adulterated agrochemicals in the markets they always went to. Similarly, all the respondents stated that the problem of agrochemical adulteration occurred in their localities, but not always. In addition, all the respondents raised that some of the products available in the market were adulterated. All the respondents reported that they had heard others complaining about the adulteration of agrochemical products they bought from the market. None of the respondents were aware of any certification or labeling system for genuine agrochemical products; they just buy products, trusting the retailers. Besides, none of them used a testing service to verify the quality of the agrochemicals they purchase. They discovered the issues with the agrochemical products after use.

#### Impact of agrochemical adulteration.

**Fig 2:**
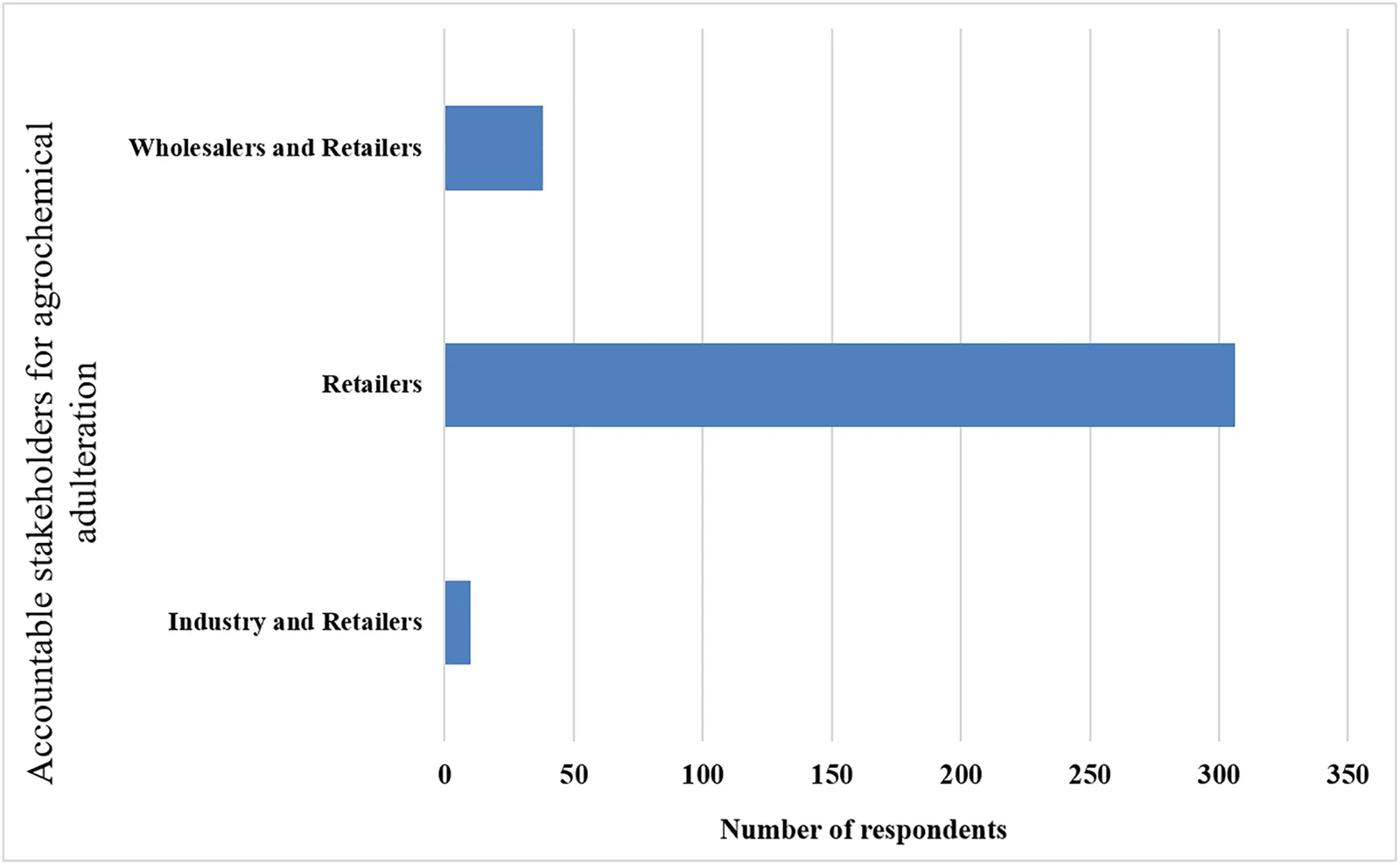
Stakeholders who were identified by farmer respondents as being accountable for the adulteration of agrochemical products.

The respondents held retailers, wholesalers, or producers accountable for the adulteration of agrochemicals. A significant proportion of respondents held retailers accountable for agrochemical adulteration (χ2 = 452, p < 0.0001) (Fig 2).

The high demand for cheaper products, corrupted supply chain, lack of monitoring, and weak regulation were the reasons for agrochemical adulteration cited by respondents (χ2 = 48, p < 0.0001). For the majority of the respondents (21%), the combination of weak regulations and high demand for cheaper products was the driving factor for agrochemical adulteration (Table 1).

**Table 1:** Respondents’ views on the causes of agrochemical adulteration and the body to which they reported adulteration.

What are the causes of agrochemical adulteration?	N	% of Total
High demand for cheap products and corruption in the supply chain	49	13.84
Lack of monitoring and corruption in the supply chain	19	5.37
Lack of monitoring and high demand for cheap products	64	18.08
Lack of monitoring, high demand for cheap products, and corruption in the supply chain	43	12.15%
Weak regulation and corruption in the supply chain	35	9.89
Weak regulation and high demand for cheap products	76	21.47
Weak regulation, high demand for cheap products, and corruption in the supply chain	68	19.21
**To whom did you report agrochemical adulterations?**		
Agrochemical suppliers	29	8.19
Agrochemical suppliers and extension service providers	9	2.54
Agrochemical suppliers and Farmer cooperatives	27	7.63
Farmer cooperative	55	15.54
Farmers’ cooperative and extension service providers	9	2.54
Farmers’ cooperative and extension service providers	1	0.28
Local authorities	141	39.83
Local authorities and agrochemical suppliers	16	4.52
Local authorities and farmers’ cooperative	67	18.93

**Fig 3:**
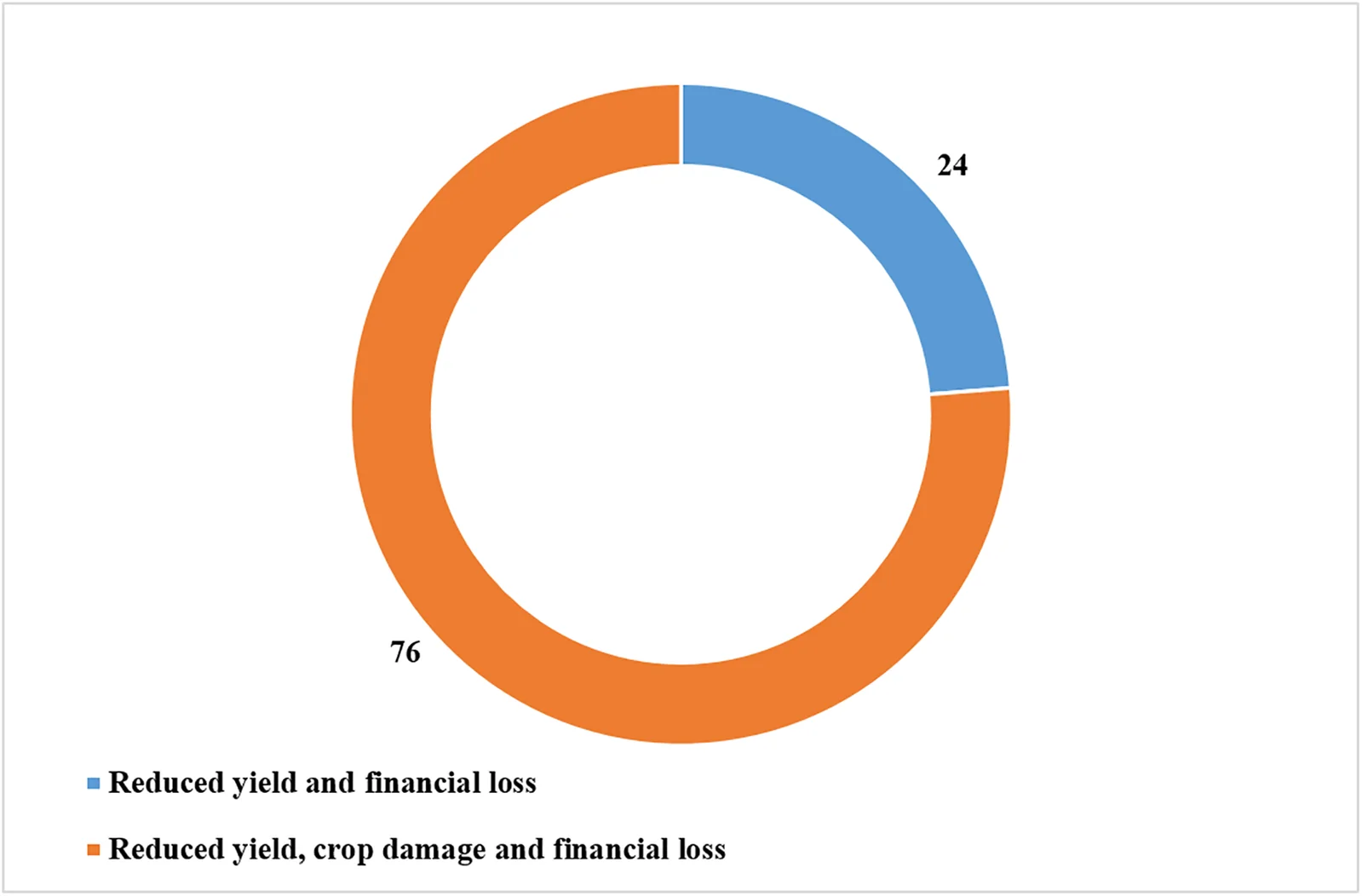
Respondents’ views on the perceived impacts of agrochemical adulteration.

The respondents agreed that agrochemical adulteration affected society. Crop damage, yield reduction, and financial loss were the impacts cited by respondents. Over two-thirds of the participants (270/354) reported a combination of reduced yield, crop damage, and financial loss as impacts of adulteration (χ2 = 97, p < 0.0001) (Fig 3). The respondents quantified the amount of produce they lost due to adulteration, and a significant variation was observed in the amount of produce lost (χ2 = 381, p < 0.0001). The majority (81%, 288/354) lost between 5 and 10 tons of their produce. Only 1% (4/354) reported a loss of more than 10 tons, and 18% (62/354) reported a loss of 1–5 tons.

To avoid purchasing adulterated products, respondents consulted extension service officers, bought from trusted retailers, or both (χ2 = 261, p < 0.0001). The majority consulted extension service officers (93%, 329), while the remaining 7% (25/354) used extension service providers and bought from trusted retailers. The respondents reported agrochemical adulteration when they encountered it. They reported this to agrochemical suppliers, extension service officers, farmers’ cooperatives, or local authorities (Table 1).

### Development agents

#### Demographic characteristics of development agents.

A total of 18 DAs, three from each Kebele, participated in the survey. Four females and 14 males DAs participated in the survey. The majority of participants, 72.2% (13/18), fell within the age range of 30–39, and 27.8% (5/18) were between 40 and 49 years of age. Half of the respondents had 1–10 years of experience, and the remaining had 11–20 years of experience. 55.6% (10/18) and 44.4% (8/18) of the respondents had a degree and diploma, respectively. Respondents’ areas of expertise were plant science (33.3%; 6/18), natural resource management (22.2%; 4/18), animal science and veterinary (16.7%; 3/18), and land/soil (11.1%; 2/18).

#### Agrochemical adulteration status, impacts, and solutions.

The DAs who participated in the survey reported that they are all aware of agrochemical adulteration (100%, 18/18). They also reported that they provided training to farmers about agrochemical adulteration. In addition, all participants confirmed that the problem of agrochemical adulteration occurs sometimes in their locality, and they sometimes hear complaints from farmers. The majority of the respondents, 83.3% (15/18), reported that dilution, reduced weight and expired products were the major areas of concern. While 11.1% (2/18) the respondents reported that dilution and reduced weight and a respondent additionally reported mixing with something as the major types of adulteration. Herbicides, fungicides and insecticides were the most adulterated agrochemicals reported by the respondents (18/18). Half of the respondents, 50% (9/18), believed that retailers are responsible for agrochemical adulteration, 27.8% (5/18) made wholesalers and retailers accountable, and 11.1% (2/18) made the industry, wholesalers and retailers accountable.

Yield reduction, unnecessary cost, resistance development, pollution and reduced farmers’ trust in agrochemicals were the major negative impacts DAs raised. Improving regulatory action, product quality screening before distribution, taking legal action on adulterators and raising awareness for farmers on the proper use and identification of adulterated agrochemicals were solutions proposed by DAs.

### Farmers’ cooperative workers

#### Demographic characteristics and status of agrochemical adulteration.

Twelve farmers’ cooperative workers (two females and ten males) participated in the study. Half of the respondents were within the 30–39 age range, 41.7% (5/12) were 40–49, and 8.3% (1/12) were in the 20–29 age range. 41.7% (5/12) of the respondents had 11–20 years of experience. 33.3% and 25% had 21–30 and 1–10 years of experience, respectively. 58.2% (7/12) had a diploma, while 41.7% (5/12) had a certificate.

All respondents 100% (12/12) reported that they distributed single fertilizer, blended fertilizer, herbicides, fungicides and insecticides for farmers. They also reported that they get the agrochemical products from a government organization. All had awareness of agrochemical adulteration and heard complaints from farmers. The majority (66.7%; 8/12) reported that herbicides and fungicides were more adulterated, whereas 25% (3/12) reported that herbicides, fungicides and insecticides, and 8.3% (1/12) reported that fungicides and insecticides were more adulterated agrochemicals. None of the respondents ever encountered a bad product.

#### Types of agrochemical adulteration, impacts, and solutions.

The majority of the respondents, 58.3% (7/12), believed that dilution, reduced weight and expired products were the major types of agrochemical adulteration. In contrast, the rest 41.7% (5/12) of them believed that dilution and reduced weight as the major types of agrochemical adulteration. Half of them, 50% (6/12), reported that retailers are responsible for agrochemical adulteration, whereas 33.3% (4/12) of them reported that wholesalers and retailers are responsible, and the rest, 16.7% (2/12), reported that the industry and retailers are responsible for agrochemical adulteration. Two-thirds of them, 66.7% (8/12), suggested that adulteration could be minimized by regular monitoring and by purchasing from known sources. In contrast, one-third, 33.3% (4/12), suggested checking the products before receiving, regular monitoring, and purchasing from known sources.

Two-thirds of the respondents (66.7%; 8/12) reported that yield reduction, unnecessary cost, and reduced farmers’ trust in agrochemicals were the major negative impacts of agrochemical adulteration, whereas one-third (33.3%; 4/12) of the respondents mentioned resistance development in addition to the above mentioned impacts. Improving regulatory action, checking the quality before distribution, taking legal action, and creating awareness among farmers on the proper use and identification of adulterated agrochemicals were the solutions proposed by the workers.

## Discussion

The number of male participants was greater than that of female participants. This imbalance may bias the results toward the perspectives of male farmers, and the experiences and views of female farmers may differ. Most of the respondents were aged 40–49 years of age. Regarding farmers’ experience in farming, 11–20 years of experience was the most common. Fertilizers, fungicides, herbicides, and insecticides were the most commonly used by all participating farmers. This implies that farmers had access to agrochemicals in their localities, which made adulteration a potential threat. All the farmers confirmed that they had the opportunity to receive training on agrochemical usage. DAs reported that they provided training to farmers. This was very high compared to the report by [5], where only approximately 54% of the participants had the opportunity for training in Tigray. An earlier report by [18] also showed very low rates, less than one-fourth. However, getting training does not always guarantee practice. For instance [19], reported that farmers’ claims of pesticide knowledge mismatch with the improper practice of pesticide storage, application, and disposal.

The farmers were all aware of agrochemical adulteration, and they obtained this information from training, extension service providers, peer discussions, and personal observation. Similar to the farmers, all the DAs and cooperative workers were well aware of the adulteration. Agricultural extension services are crucial for creating sustainable agriculture by introducing farmers to new technologies and promoting safe crop production [20,21]. The understanding of farmers is the first step to avoiding the use of adulterated products. In this regard, farmers’ understanding could be an indicator of the efforts to create awareness about agrochemical adulteration, which would pave the way to minimize the availability of adulterated products in the market. The farmers also reported the unintentional use of adulterated products. Although farmers claimed that they could differentiate between adulterated and non-adulterated agrochemicals, the vast majority were not certain about their capability to identify adulterated products. This could indicate the presence of adulterated products in the markets and the difficulty of identifying adulterated products, despite understanding adulteration. Age, experience, and educational background did not significantly predict farmers’ ability to identify adulterated products, which implies the complexity of the issue that farmers experience, and secondary education cannot resolve. In reality, distinguishing between adulterated and non-adulterated products can be challenging unless specific quality assurance testing is performed [22]. reported that farmers cannot distinguish genuine products from adulterated ones [15]. also reported farmers’ challenges in detecting adulterated pesticides.

Low effectiveness during use, packaging, and weight were the means farmers used to identify adulterated products. Realizing that a product was adulterated after usage is a double jeopardy, where farmers lose their money to purchase the adulterated product and their crop as well, due to reduced effectiveness. Farmers often rely on inaccurate methods to identify adulterated products [23]. Dilution and weight reduction were the most commonly observed types of adulteration reported by farmers. DAs and cooperative workers also reported dilution, reduced weight, and mixing with something else. Adulteration may not always be intentional; manufacturing errors can lead to adulteration [12]. Retailers might unintentionally sell adulterated products; if it is intentional, they might consider easily undetectable methods, such as dilution. Identifying weight reduction may be possible; however, dilution may be difficult.

All farmers who participated reported that they had unintentionally purchased adulterated agrochemical products. Fungicides and herbicides are often adulterated in the area. DAs also reported insecticides, fungicides, and herbicides as the most commonly adulterated products. However, other researchers have shown adulteration in other products, such as fertilizers. Adulteration and counterfeiting are reported to be uncommon in urea fertilizers [12]. Farmers reported hearing complaints from other farmers about adulterated products. DAs and cooperative workers also heard complaints about adulteration by farmers. Users had no information about the verification or labeling of genuine products and often relied on retailers. Relying on retailers to obtain genuine products did not seem to work. This is in agreement with the report by [24]. Even though farmers mistrust retailers, they continued to use the products available in the market, which could be due to the lack of an alternative market.

Farmers, DAs, and cooperative workers held retailers, wholesalers, and producers accountable for adulterating agrochemicals. Similarly, food adulteration is caused by dishonest producers and traders [25]. According to the respondent farmers, the need for cheaper alternatives, corruption in the supply chain, lack of monitoring, and weak regulation were the reasons for the observed adulteration in the area. It is obvious that people prefer cheaper products, mainly those that they frequently buy. The lack of monitoring and weak regulation could be due to the lack of professionals in the area, and shops are scattered and difficult to monitor. Ethiopia has a pesticide regulation law; however, the enforcement of the law is limited due to the shortage of trained human resources, lack of standard testing laboratory, and poor monitoring in the value chain [26,27]. Large numbers of informal traders sell pesticides and fertilizers without licenses, training, and compliance requirements [28]. There is no functional post-registration monitoring, which contributes to adulterated agricultural inputs in the market [29]. According to [28], products without any labeling or names on the containers were observed in different retail shops in the country.

All participants, including DAs and cooperative workers, agreed on the negative impact of adulteration on society. Crop damage, yield reduction, reduced farmers’ trust in agrochemicals, resistance development and financial loss were reported [15]. reported that farmers agreed to the impacts of adulterated products on human and animal health and the environment. The impact does not stop at buying adulterated products; these adulterated products cannot function as intended. Consequently, crops may fail, or the yield may be very low [12]. reviewed the negative impact of applying adulterated products on crop growth. Farmers sought support from extension officers or bought from known retailers to minimize the possibility of purchasing adulterated products. This was also suggested by DAs and cooperative workers. Although the support farmers received from extension service providers helped minimize the purchase of adulterated products, identifying adulterated products might not be an easy task for extension service officers. As it requires resources and special analytical techniques [30]. In addition, retailers may stock adulterated products unknowingly due to poor regulation, as reported in food adulteration by [31]. Farmers reported adulteration to various stakeholders as they encountered, such as retailers/wholesalers, extension officers, farmers’ cooperatives, and other local authorities. The reporting was informal; there was no formal system and procedure to follow to report and claim justice.

To address the problem, DAs and cooperative workers suggested strengthening regulation and quality control, taking legal actions, and raising awareness among farmers. Although awareness and knowledge were desirable, the practice of reducing adulteration through legal procedures and actions remained difficult to implement. This study provided insight into the status of agrochemical adulteration and could function as a foundation for future work. However, no market investigation for adulterated products was made, which could be one of the future works.

A complete homogeneity was observed in farmers’ use of agrochemicals (fertilizer, insecticide, fungicide, and herbicide), participation in agrochemical-use training, and awareness of agrochemical adulteration. Therefore, these results should be interpreted in the context of the study area, Wolmera woreda, where farmers are likely to have similar exposure due to a wide distribution of agrochemicals and the implementation of extension services.

## Conclusion and recommendation

### Conclusion

This study aims to survey farmers, DAs, and farmers’ cooperative workers’ perspectives on the agrochemical adulteration, status in their localities, and its impacts on the agriculture sector. The results revealed that all stakeholders have a good understanding of agrochemical adulteration and reported that they encountered adulterated products. The majority of the farmers were not certain about their capability to identify adulterated products. Low effectiveness during use, packaging, and weight were the means used to identify adulterated products. Dilution and weight reduction were reported as the most commonly observed types of adulteration. Fungicides, herbicides, and insecticides were reported as the most commonly adulterated products. Retailers, wholesalers, and producers were held accountable for adulterating agrochemicals. The need for cheaper alternatives, corruption in the supply chain, lack of monitoring, and weak regulation were the reasons for the adulteration observed in the area. The negative impact of adulteration on society includes crop damage, yield reduction, reduced farmers’ trust in agrochemicals, resistance development, and financial loss. These findings play a role in a better understanding of the status of adulteration in the area, farmers’ understanding of adulteration, and the intervention needed. To the best of our knowledge, this work is novel and could contribute valuable information on the status of agrochemical adulteration and possible ways to monitor and manage adulteration.

### Recommendation

- Future studies should assess product authenticity using standardized analytical and laboratory based methods.
- Regulatory authorities should establish a technology driven and comprehensive quality monitoring system for both locally produced and imported products across the entire value chain and maintain accountability.
- Routine and risk based inspections of agrochemical products should be conducted, particularly at storage facilities and warehouses.
- Farmers should be systematically trained and supported to identify counterfeit or substandard products using practical, field-based verification methods.

## Supporting information

S1 Data Data. (XLSX)
